# Urban noise measurements in the City of Buenos Aires during the mandatory quarantinea)

**DOI:** 10.1121/10.0002423

**Published:** 2020-11-30

**Authors:** Germán Said, Agustín Arias, Leonardo Carilli, Agustín Stasi

**Affiliations:** Acoustic Impact Department, Environmental Protection Agency, Buenos Aires, C1073AAW, Argentina

## Abstract

During preventive and mandatory social isolation decreed by the Argentine National Government to contain the spread of COVID-19, the city of Buenos Aires has experienced a marked decrease in vehicular traffic. To study this new scenario, the Acoustic Impact Department of the Environmental Protection Agency carried out a series of uninterrupted urban noise measurements for one week in five measurement points. The results were compared with those obtained before quarantine and with the maximum permissible limits according to current legislation. Although considerable decreases in sound energy have been obtained, it was not possible to determine global values of noise level reduction since the results were of different magnitudes in each location and period.

## I. INTRODUCTION

In its January 2019 newsletter, the International Commission for Acoustics (ICA), in collaboration with “La Semaine du Son” (LSdS) association, announced that 2020 was declared the International Year of Sound (IYS 2020).^1^ This initiative was born from the approval of UNESCO Document 39 C/49 through Resolution 39 C/59 at its assembly on November 13, 2017, on the “Importance of sound in today's world: Promotion of good practices.”^2,3^ Among its key points, the following stands out: *“The sound environment reflects and shapes our individual and collective behavior, and our productivity and capacity to live in harmony together. Giving more importance to sound-related issues in our increasingly noisy world has thus become a vital matter.”*

In this context, ICA began promoting events around the world, as well as congresses and meetings organized by its international affiliates. However, these activities had to be postponed due to the pandemic unleashed by the worldwide spread of COVID-19, which forced states and nations to take measures of confinement, social distancing, border closure, and cessation of activities to minimize the risks of contagion. This new reality, as unexpected as forced, highlighted the importance of the UNESCO key point mentioned above.

The Environmental Protection Agency of the city of Buenos Aires (APrA, for its Spanish acronym), promoted the realization of a comparative study between urban noise levels before and during the quarantine decreed by the national government throughout the Argentine territory as of March 20, 2020. This study was performed and promoted within the framework of the *Week of Sound*, commemorated the last Wednesday of each April.

## II. LEGAL FRAMEWORK

The Autonomous City of Buenos Aires is the capital of the Argentine Republic. Its surface is 203 km^2^ and its population is estimated to be 2 890 151 inhabitants, according to the results of the 2010 census.^4^

In 2004, the City Legislature passed Law No. 1540, entitled “Control of Noise Pollution in the Autonomous City of Buenos Aires.”^5^ In 2007, the mayor of the city dictated the regulation of Law No. 1540 through Decree No. 740, which establishes, among others, the technical guidelines for carrying out a permanent program to measure noise levels outdoors, in areas of great urban concentration considered as the most affected by noise pollution.^6^ It also establishes the maximum permissible limits of each outdoor acoustic sensitivity area (OASA) for both day-time (from 07:01 to 22:00) and night-time (from 22:01 to 07:00). These limits, listed in Table I, are directly linked to land use, identifying residential, commercial, industrial, historic protection areas, and nature reserves, among others. Finally, in its Annex XI, the technical guidelines for the creation of the city noise maps are established, using the L_day_ and L_night_ indicators established in the ISO 1996–2 standard.^7^

**TABLE I.** OASA. Law No. 1540 and Decree No. 740 of the City of Buenos Aires.

Outdoors			
Acoustic sensitivity area	Maximum permissible limits		Characteristic zoning
	Leq dB(A) Day-time	Leq dB(A) Night-time	
Type I	60	50	Ecological reserve
Type II	65	50	Residential
Type III	70	60	Commercial and economic
Type IV	75	70	Industrial
Type V	80	75	Primary road networks

## III. METHODS

Currently, the Environmental Protection Agency is the enforcement authority of Law No. 1.540, and its Acoustic Impact Department is in charge of monitoring noise levels on public roads. Various projects have emerged in this area, where the preparation of the first strategic noise map of the city, completed in May 2018, stands out.^8^ The results of this work were validated with at least one week long measurements in 162 microphone locations, using five mobile monitoring stations. A permanent noise monitoring network is planned to be installed in the next few years.

For this study, the first step was to select the five measurement points throughout the City, in order to cover different scenarios. To enable precise comparisons between urban situations, the five points to choose should have been previously used for validating the city noise map. One of the premises for that task was that the computational model error is more significant in streets with reduced flow since traffic speed and counting sensors are commonly placed on relevant arteries, and the results for less relevant streets are generated by statistical calculations. For this reason, the vast majority of those 162 points were located on avenues. Based on these considerations, the technical team drew up a list of possible locations containing various main arteries that connect different city zones and their accesses. Finally, the following were chosen as interest points:

• Buenos Aires Obelisk: Iconic place in the city par excellence. In this point, the 9 de Julio avenue, which connects the north and south accesses, intersects with the Corrientes Avenue, known as “the street that never sleeps,” since it has the highest concentration of bookstores, theaters, pizza shops, and bars in Buenos Aires.
• 226 Callao Avenue: This point is three blocks from the National Congress and several banks are located in the vicinity.
• 7242 Rivadavia Avenue: A main artery that connects the city from east to west.
• 4350 Triunvirato Avenue: Located in the northwest area of the city, in the residential neighborhood of Villa Urquiza.
• 4550 Santa Fe Avenue: A main artery that connects the north of the city with the center.

Measurements were performed with five 01 dB *DUO* monitoring stations (IEC-61672 Class-1^9^) with their corresponding accessories. Although these devices were configured to self-calibrate every day using an electrical reference signal, an acoustic calibration is required at the beginning and end of the measurement week. For this, a 01 dB *Cal 21* calibrator was used (IEC 60 942 Class 1^10^). As indicated in Annex XI of Decree No. 740 of the City of Buenos Aires, the microphones were installed 4 m above the ground level. Finally, the data obtained from the measurements were processed with the analysis software, dBTrait v.5.5.2.

**FIG. 1.**
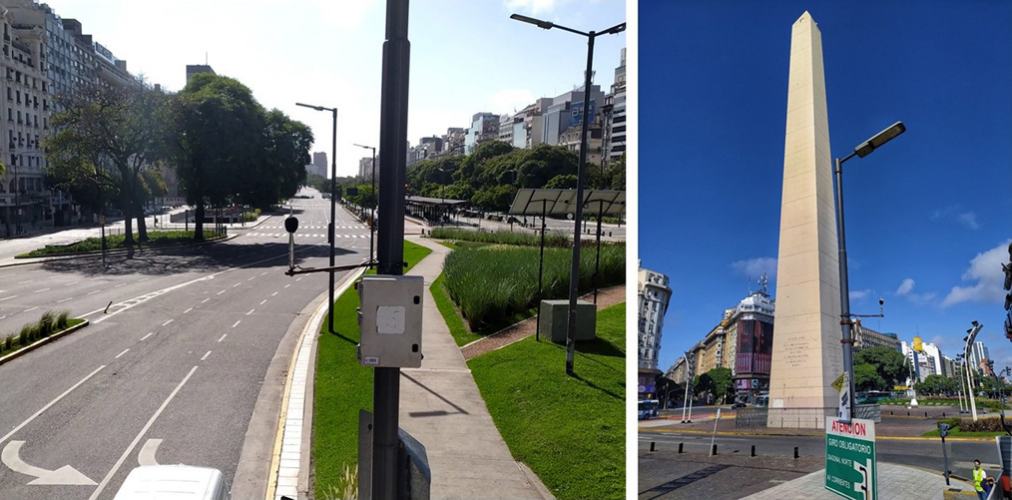
(Color online) Monitoring station on 9 de Julio Avenue, next to the Buenos Aires Obelisk.

The stations were configured to record A-weighted equivalent continuous sound pressure levels (LA_eq_) and their corresponding third-octave band data with an integration time of 10 s, which allows obtaining high temporal and spectral resolution results. In addition, a trigger was configured to record the audio signal of any event that generated LA_eq_ higher than 85 dBA, allowing the identification of sound sources, which can be eliminated later in case they were identified as abnormal cases. Figure 1 is a photograph taken after installing the monitoring station near the Obelisk on the 9 de Julio Avenue, one of the most iconic places of the country.

The measurements were performed from Monday, April 20 to Sunday, April 26. It should be noted that in the two weeks prior to the start of this study, movement and activity restrictions were more stringent. The measurements corresponding to Saturday and Sunday were discarded in order to exclusively represent the periods with the highest activity.

## IV. RESULTS

### A. Global average levels

The first evaluation carried out corresponds to the LA_eq_ for each day-time (L_day_), night-time (L_night_), and their weekly energy averages, as established in Law No. 1540 and Decree No. 740. These results are shown in Table II.

**TABLE II.** Comparison of sound levels before and during quarantine. Day-time and night-time period. Weekly energy average.

Weekly energy average								
Station location	Lday (dBA)		Day-time difference		Lnight (dBA)		Night-time difference	
	Before quarantine	During quarantine	In dB	In %	Before quarantine	During quarantine	In dB	In %
Obelisk	71.9	69.9	−2.0	−36.9%	68.3	64.4	−3.9	−59.3%
226 Callao Ave.	73.8	71.9	−1.9	−35.4%	71.8	68.0	−3.8	−58.3%
7264 Rivadavia Ave.	75.3	70.6	−4.7	−66.1%	72.9	66.0	−6.9	−79.6%
4350 Triunvirato Ave.	70.8	69.1	−1.7	−32.4%	66.6	63.9	−2.7	−46.3%
4550 Santa Fe Ave.	76.0	74.6	−1.4	−27.6%	72.8	69.6	−3.2	−52.1%

During day-time period, the maximum difference was obtained at the point of Rivadavia Avenue, reaching 4.7 dB, equivalent to a 66.1% sound energy reduction. As observed on site during equipment installation, the heavy traffic flow, characterized mainly by buses using the three central “Metrobús” exclusive lanes, has been considerably reduced on this avenue.

On the other hand, the smallest difference registered reached 1.4 dB at the point of Santa Fe Avenue, which is equivalent to a sound energy reduction of 27.6%. In this case, although light traffic decreased considerably as it could be observed *in situ*, the same did not happen with the buses that circulate in the three central “Metrobús” exclusive lanes of this avenue. However, it has not been possible to obtain vehicle count and speed data that allows establishing a precise quantitative relationship between noise and vehicle flow reductions during quarantine.

During the night period, the differences between the two situations were even greater, as expected. The maximum noise level decrease registered was about 6.9 dB in Rivadavia Avenue, equivalent to a sound energy reduction of 79.6%. The minimum difference was observed at the point of Triunvirato Avenue, reaching 2.7 dB, which corresponds to a reduction of 46.3%. However, it should be noted that the lowest noise levels were measured at this point, being 66.6 and 63.9 dBA, before and during quarantine, respectively. This suggests that the levels measured during quarantine represent a kind of noise floor at this location. It is interesting to note that with the quarantine restrictions, noise levels below 70 dBA were obtained in all five locations.

### B. Comparison with the maximum permissible limits (MPLs)

**FIG. 2.**
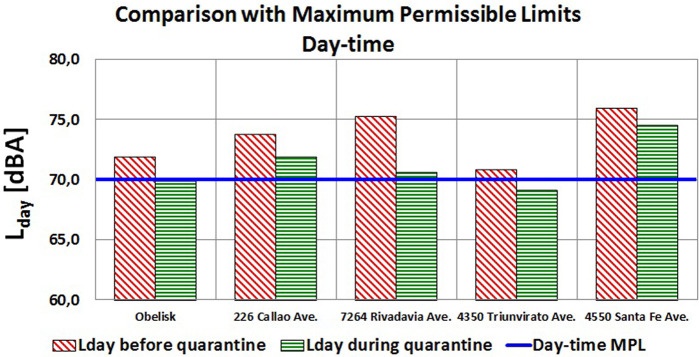
(Color online) Comparison of L_day_ noise levels with their respective MPLs.

**FIG. 3.**
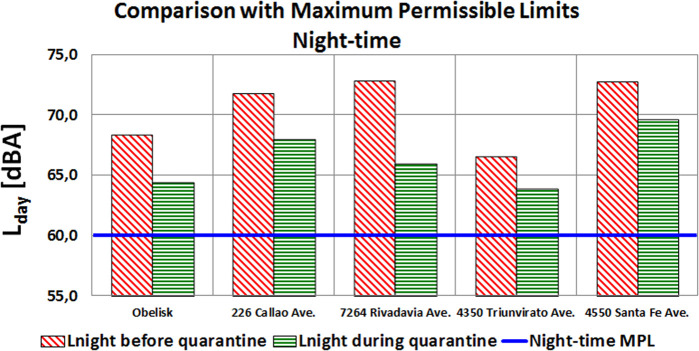
(Color online) Comparison of L_night_ noise levels with their respective MPLs.

The following analysis corresponds to the comparison between the weekly energy average levels obtained and the MPLs established in both Law No. 1540 and Decree No. 740. The five points are within type III OASA, so the limits for all of them are 70 and 60 dBA for day-time and night-time, respectively. Figures 2 and 3 show these results.

Only the day-time noise levels recorded at the Obelisk and Triunvirato Avenue were below the corresponding MPL of 70 dBA during quarantine. Sound levels at Rivadavia Avenue were near this MPL due to the drastic energy reduction.

Although there were considerable drops in levels overnight, none of the five points reached the MPL of 60 dBA, evidencing how difficult it is to comply with the legal limits on avenues, even when traffic is so restricted. That is why one of the tasks to be carried out by the Acoustic Impact Department is to review these limits based on both the results of the city noise map and long-term measurements. This statement should not be interpreted in terms of the levels recommended by the WHO.^11^

### C. Time histories of measurements

**FIG. 4.**
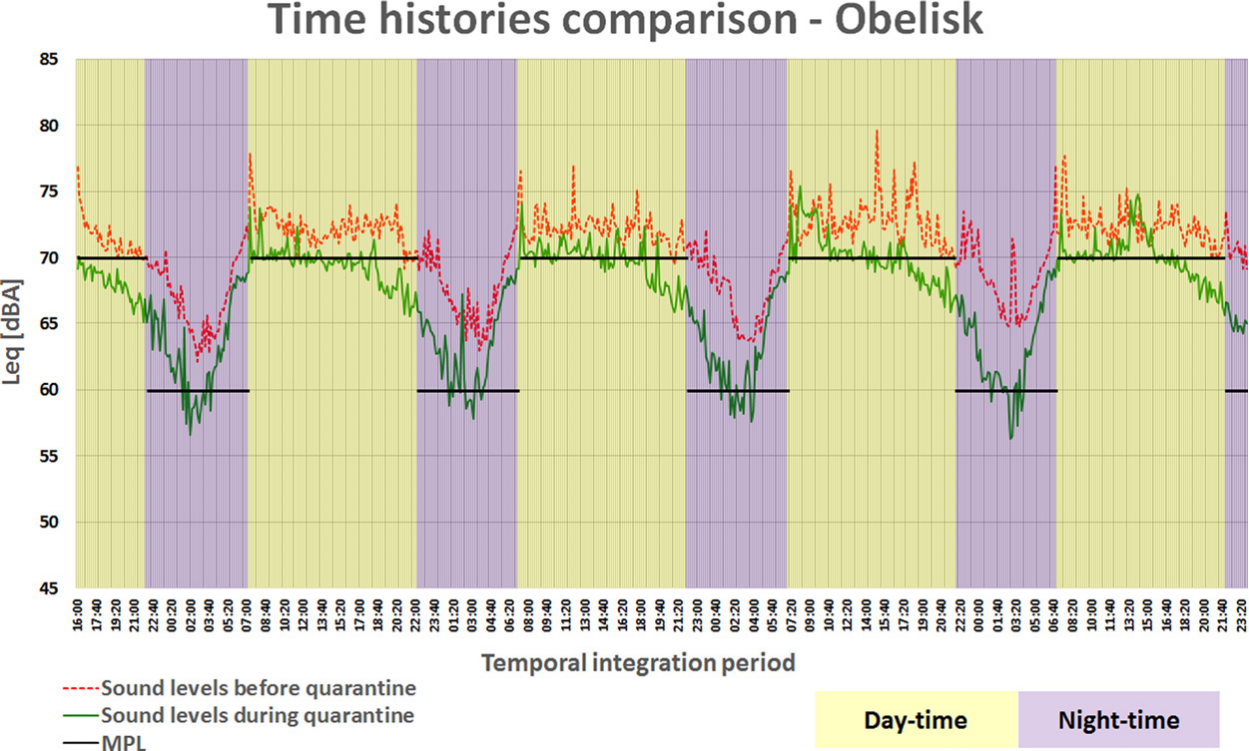
(Color online) Time histories and Maximum Permissible Limit before and during quarantine at the Obelisk.

Figure 4 shows the time histories of the measurements taken at the Buenos Aires Obelisk before and during quarantine, as well as the MPL for each period. An integration time of 15 min was used for illustrative clarity. This comparison was made for the five measurement points to look for patterns of change in traffic flow behavior.

During quarantine, sound levels below the MPL of 60 dBA were observed during deep rest hours (between 02:00 and 05:00 am) at the Obelisk and Triunvirato Avenue locations, although the former is not located in a residential area. In the case of Rivadavia Avenue, the levels for that same period were found near the MPL, while those corresponding to Callao and Santa Fe Avenues were considerably above 60 dBA.

As for the day-time period, a sound level decrease trend was observed from approximately 6:00 pm at the Obelisk and Callao Avenue. This is not evident in the measurements carried out in a normal situation, which suggests that the departure of vehicles from the central area of the City begins earlier during quarantine.

### D. Calculation of uncertainty

The results of the expanded uncertainty are in all cases between 0.8 and 0.9 dB, except for a particular case at Rivadavia Avenue before quarantine, where it jumps to 1.4 dB at night-time (one L_night_ was approximately 2.2 dB above the rest during the measurement week). A value of 0.4 for Type-B uncertainty was used according to Payne's study.^12^

## V. CONCLUSIONS

The data collected during the measurement campaign allowed some interesting conclusions to be made. However, since the samples that were taken cover specific points, it would not be correct to make widely generalized statements about the sound reality of the City of Buenos Aires in times of quarantine. A clear example of this is the uneven variations in the results and differences obtained at each of the five locations.

No levels were observed that comply with the MPL of 60 dBA for the night-time. However, in a more detailed analysis, at Obelisk and Triunvirato Avenue, the sound levels registered were below 60 dBA between 02:00 and 05:00 am (deep rest) during quarantine. This analysis, together with the results of the city noise map, is the basis for future work to review these limits and propose new ones, which should be adjusted to what is really achievable.

Another interesting analysis emerges from the measurements made on Santa Fe Avenue, where the great contribution that buses make to urban noise is evidenced. During quarantine, a weekly average L_day_ of 74.6 dBA was obtained, just 1.4 dB below the 76.0 dBA registered in the measurements carried out previously. This situation is exclusively attributed to the little variation observed in bus flow. However, vehicle flow and speed data are needed to perform an objective analysis of this situation.

The data corroborates that one of the main sources of environmental noise in the City of Buenos Aires is vehicular transportation. It is difficult to imagine a similar situation that would allow us to know the “hypothetical” levels that would exist if vehicle flows were greatly reduced. This experience shows how complex it is to achieve a decrease in significant sound level and should promote awareness of the problem of urban noise in large cities. Governments and associations must work together to carry out educational and good practices campaigns, as well as promote and implement concrete noise mitigation actions in order to improve the quality of life for people.
